# Supplementary material for: Freshwater Mussel Viromes Increase Rapidly in Diversity and Abundance When Hosts Are Released from Captivity into the Wild
Source: Animals (Basel). 2024 Aug 30;14(17):2531. doi: 10.3390/ani14172531 (PMC11393864; doi:10.3390/ani14172531)
Supplement: Supplementary file 1 [file animals-14-02531-s001.zip › Supplemental Figures V2.0.pdf]

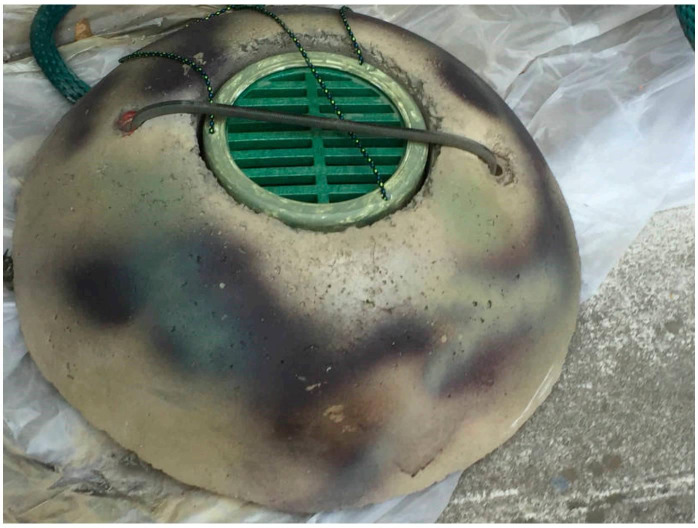

Supplemental Figure S1. Image showing an example of a concrete silo used to hold freshwater mussels at research sites. Photo credit: Julie Campbell, U.S. Fish and Wildlife Service (USFWS). Image used under public domain availability from the USFWS NCTC Image Library.

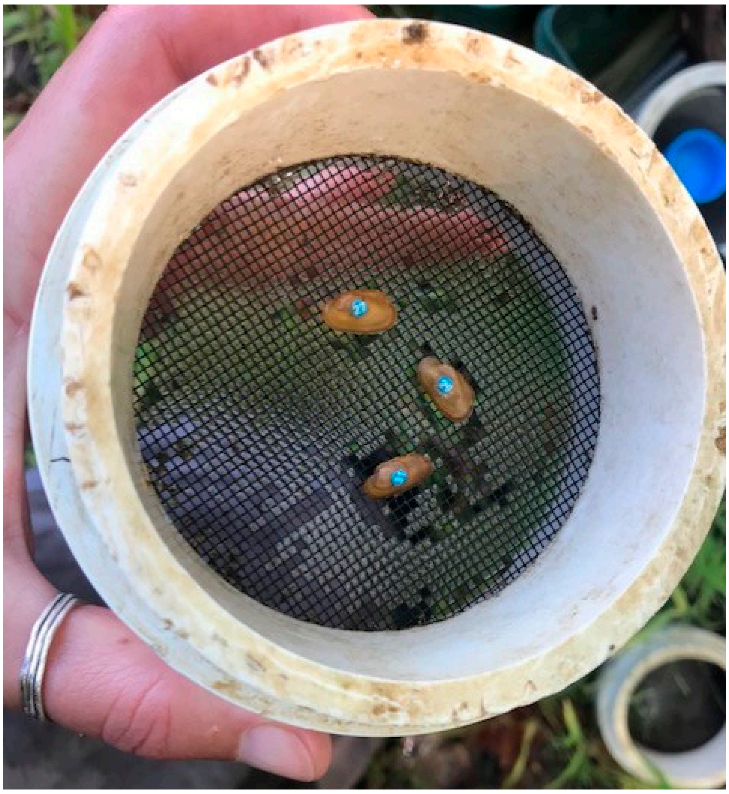

Supplemental Figure S2. Image showing juvenile mussels within the inner chamber of the silos used in the Upper Tennessee River Basin mussel virome study. Juveniles were enclosed in polyvinyl chloride cups with mesh screens, which were anchored in the center of concrete mussel silos and placed on river beds at study sites. Photo credit: Rose Agbalog, USFWS.

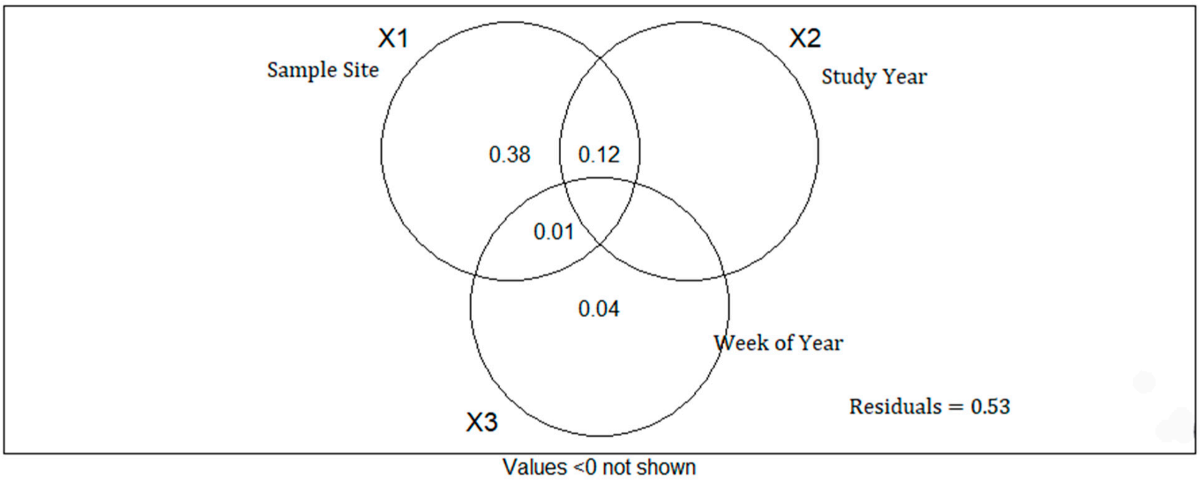

Supplemental Figure S3. Variation partitioning plot showing the coefficient of determination (R^2) adjusted values and overlap between the variables sample site, study year, and week of year in explaining observed variation in juvenile freshwater mussel viromes from the Upper Tennessee River Basin.
